# IMPROVING COGNITION IN OLDER CANCER SURVIVORS USING NONPHARMACOLOGIC INTERVENTIONS: A SYSTEMATIC REVIEW

**DOI:** 10.1093/geroni/igac059.2265

**Published:** 2022-12-20

**Authors:** Jennifer Blackwood, Abigail Simone

**Affiliations:** University of Michigan-Flint, Flint, Michigan, United States; University of Michigan--Flint, Flint, Michigan, United States

## Abstract

Cancer-related cognitive dysfunction (CRCD) is a side-effect of chemotherapy, particularly among young and adult populations. Results indicate CRCD is also prevalent in older adult populations. While various non-pharmacological interventions for cognitive impairments have been studied in young cancer and older adult populations, limited information is available regarding non-pharmacologic interventions for older adults with cancer. The purpose of this systematic review is to describe the current non-pharmacologic interventions for CRCD in the older adult cancer population. Databases searched included PubMed, MEDLINE, CINAHL, and EMBASE. Articles meeting inclusion criteria were appraised by 2 reviewers independently. The Cochrane Risk of Bias Assessment was used to assess study quality. The search located 3441 articles; 4 met inclusion criteria. Cognitive domains assessed by included studies comprised executive function (n=2), attention (n=1), learning/memory (n=2), perceptual-motor (n=1), and a general measure of global cognitive function (n=3). Two studies used exercise interventions and 2 employed cognitive training interventions. One exercise intervention improved executive function, while attention and learning/memory improved following cognitive training. However, a limited number of studies utilizing non-pharmacological approaches for treating cognitive impairment in this population showed high methodological heterogeneity. Non-pharmacologic interventions demonstrated positive outcomes for CRCD, however, methodological concerns in the included studies prevented definitive recommendations from being made. Findings may guide additional studies needed in this field in order to make more robust conclusions.

